# *Escherichia coli* Sequence Type 410 Is Causing New International High-Risk Clones

**DOI:** 10.1128/mSphere.00337-18

**Published:** 2018-07-18

**Authors:** Louise Roer, Søren Overballe-Petersen, Frank Hansen, Kristian Schønning, Mikala Wang, Bent L. Røder, Dennis S. Hansen, Ulrik S. Justesen, Leif P. Andersen, David Fulgsang-Damgaard, Katie L. Hopkins, Neil Woodford, Linda Falgenhauer, Trinad Chakraborty, Ørjan Samuelsen, Karin Sjöström, Thor B. Johannesen, Kim Ng, Jens Nielsen, Steen Ethelberg, Marc Stegger, Anette M. Hammerum, Henrik Hasman

**Affiliations:** a Department of Bacteria, Parasites and Fungi, Statens Serum Institut, Copenhagen, Denmark; b Department of Clinical Microbiology, Hvidovre University Hospital, Hvidovre, Denmark; c Department of Clinical Medicine, Faculty of Health and Medical Sciences, University of Copenhagen, Copenhagen, Denmark; d Department of Clinical Microbiology, Aarhus University Hospital, Aarhus, Denmark; e Department of Clinical Microbiology, Slagelse Hospital, Slagelse, Denmark; f Department of Clinical Microbiology, Herlev and Gentofte Hospital, Herlev, Denmark; g Department of Clinical Microbiology, Odense University Hospital, Odense, Denmark; h Department of Clinical Microbiology, Rigshospitalet, Copenhagen, Denmark; i Department of Clinical Microbiology, Aalborg University Hospital, Aalborg, Denmark; j Antimicrobial Resistance and Healthcare Associated Infections (AMRHAI) Reference Unit, National Infection Service, Public Health England, London, United Kingdom; k Institute of Medical Microbiology, Justus Liebig University Giessen and German Centre for Infection Research (DZIF), Partner Site Giessen-Marburg-Langen, Giessen, Germany; l Norwegian National Advisory Unit on Detection of Antimicrobial Resistance, Department of Microbiology and Infection Control, University Hospital of North Norway, Tromsø, Norway; m Department of Pharmacy, Faculty of Health Sciences, UiT-The Arctic University of Norway, Tromsø, Norway; n Public Health Agency of Sweden, Stockholm, Sweden; o Department of Infectious Disease Epidemiology and Prevention, Statens Serum Institut, Copenhagen, Denmark; JMI Laboratories

**Keywords:** BEAST, epidemiology, Escherichia coli, outbreak, evolution, high-risk clone

## Abstract

Escherichia coli sequence type 410 (ST410) has been reported worldwide as an extraintestinal pathogen associated with resistance to fluoroquinolones, third-generation cephalosporins, and carbapenems. In the present study, we investigated national epidemiology of ST410 E. coli isolates from Danish patients. Furthermore, E. coli ST410 was investigated in a global context to provide further insight into the acquisition of the carbapenemase genes *bla*_OXA-181_ and *bla*_NDM-5_ of this successful lineage. From 127 whole-genome-sequenced isolates, we reconstructed an evolutionary framework of E. coli ST410 which portrays the antimicrobial-resistant clades B2/H24R, B3/H24Rx, and B4/H24RxC. The B2/H24R and B3/H24Rx clades emerged around 1987, concurrently with the C1/H30R and C2/H30Rx clades in E. coli ST131. B3/H24Rx appears to have evolved by the acquisition of the extended-spectrum β-lactamase (ESBL)-encoding gene *bla*_CTX-M-15_ and an IncFII plasmid, encoding IncFIA and IncFIB. Around 2003, the carbapenem-resistant clade B4/H24RxC emerged when ST410 acquired an IncX3 plasmid carrying a *bla*_OXA-181_ carbapenemase gene. Around 2014, the clade B4/H24RxC acquired a second carbapenemase gene, *bla*_NDM-5_, on a conserved IncFII plasmid. From an epidemiological investigation of 49 E. coli ST410 isolates from Danish patients, we identified five possible regional outbreaks, of which one outbreak involved nine patients with *bla*_OXA-181_- and *bla*_NDM-5_-carrying B4/H24RxC isolates. The accumulated multidrug resistance in E. coli ST410 over the past two decades, together with its proven potential of transmission between patients, poses a high risk in clinical settings, and thus, E. coli ST410 should be considered a lineage with emerging “high-risk” clones, which should be monitored closely in the future.

**IMPORTANCE** Extraintestinal pathogenic Escherichia coli (ExPEC) is the main cause of urinary tract infections and septicemia. Significant attention has been given to the ExPEC sequence type ST131, which has been categorized as a “high-risk” clone. High-risk clones are globally distributed clones associated with various antimicrobial resistance determinants, ease of transmission, persistence in hosts, and effective transmission between hosts. The high-risk clones have enhanced pathogenicity and cause severe and/or recurrent infections. We show that clones of the E. coli ST410 lineage persist and/or cause recurrent infections in humans, including bloodstream infections. We found evidence of ST410 being a highly resistant globally distributed lineage, capable of patient-to-patient transmission causing hospital outbreaks. Our analysis suggests that the ST410 lineage should be classified with the potential to cause new high-risk clones. Thus, with the clonal expansion over the past decades and increased antimicrobial resistance to last-resort treatment options, ST410 needs to be monitored prospectively.

## Abstract (precis)

Extraintestinal pathogenic Escherichia coli (ExPEC) is the main cause of urinary tract infections and septicemia. Significant attention has been given to the ExPEC sequence type ST131, which has been categorized as a “high-risk” clone. High-risk clones are globally distributed clones associated with various antimicrobial resistance determinants, ease of transmission, persistence in hosts, and effective transmission between hosts. The high-risk clones have enhanced pathogenicity and cause severe and/or recurrent infections. We show that clones of the E. coli ST410 lineage persist and/or cause recurrent infections in humans, including bloodstream infections. We found evidence of ST410 being a highly resistant globally distributed lineage, capable of patient-to-patient transmission causing hospital outbreaks. Our analysis suggests that the ST410 lineage should be classified with the potential to cause new high-risk clones. Thus, with the clonal expansion over the past decades and increased antimicrobial resistance to last-resort treatment options, ST410 needs to be monitored prospectively.

## INTRODUCTION

Escherichia coli sequence type 131 (ST131) is a high-risk clone, defined as (i) globally distributed, (ii) associated with multiple antimicrobial resistance determinants, (iii) able to colonize and persist in hosts for more than 6 months, (iv) capable of effective transmission between hosts, (v) having enhanced pathogenicity and fitness, and (vi) able to cause severe and/or recurrent infections (1). ST131 is associated with a steady increase in antimicrobial resistance globally, including fluoroquinolone, third-generation cephalosporin, and carbapenem resistance (2–4). A recent study of 10 extended-spectrum β-lactamase (ESBL)-producing E. coli ST410 isolates from Germany provided preliminary evidence that this lineage includes a new successful clone with cross-sectorial transmission between wildlife, humans, companion animals, and the environment (5, 6). This high-risk potential is supported by two recent reports from China (7) and Italy (8) of ST410 isolates carrying the acquired carbapenemase gene *bla*_OXA-181_. Similarly, ST410 carrying *bla*_OXA-181_ has also been shown to be involved in a small hospital outbreak in Denmark (9). Recent complete-genome sequencing of an ST410 isolate from a Danish patient further revealed a multidrug-resistant strain with two carbapenemase genes (*bla*_OXA-181_ and *bla*_NDM-5_) carried on IncX3 and IncF plasmids, respectively (10). Several studies on molecular characterization of carbapenemase-producing *Enterobacteriaceae* (CPE) among inpatients have indicated clonal spread of CPE from patient to patient (11, 12). Carbapenemase genes are often located on mobile elements containing multiple resistance genes (13, 14), leading to potential horizontal transfer to other bacterial species. The mortality associated with invasive infections caused by CPE is high (15), making the spread of CPE an immense clinical concern. As ST410 has been increasingly reported worldwide, it is important to determine whether the ST410 lineage is causing new pandemic high-risk clones similar to ST131.

In the current study, we investigated the epidemiology of third-generation cephalosporin- and carbapenem-resistant ST410 E. coli isolates from Danish patients, to elucidate whether multidrug-resistant ST410 was causing national outbreaks or, alternatively, if a global clone was being introduced multiple times. We further aimed to set ST410 into a global context by the addition of genome data from other national collections as well as from publicly available genomes extracted from the EnteroBase (http://enterobase.warwick.ac.uk) database to provide further insight into the acquisitions of the carbapenemase genes *bla*_OXA-181_ and *bla*_NDM-5_ of this successful lineage.

(The data were partly presented at DANMAP 2014, DANMAP 2015, and DANMAP 2016.)

## RESULTS

### Genotypic characterization.

Genotypic characterization was applied to the 127 E. coli ST410 genomes included in the study (49 genomes, collected from 46 patients from the national Danish surveillance program DANMAP, and 78 international genomes), including whole-genome sequencing (WGS)-based resistance gene profiling, subtyping by *fimH* allelic variation, and identification of plasmid replicons and plasmid multilocus sequence typing (pMLST) subtypes (see Table S1 in the supplemental material).

10.1128/mSphere.00337-18.10 TABLE S1  Extended isolate characterization. Download TABLE S1, XLSX file, 0.03 MB. Copyright © 2018 Roer et al. 2018 Roer et al. This content is distributed under the terms of the Creative Commons Attribution 4.0 International license.

Of the 127 ST410 genomes, 114 genomes contained at least one of 14 different ESBL genes, plasmid-mediated AmpC (pAmpC) genes, or carbapenemase genes or a truncated pAmpC gene, whereas no gene was detected in 13 genomes (Table 1). The majority of the genomes carried *bla*_CTX-M-15_ (66%), followed by *bla*_OXA-181_ (53%), *bla*_CMY-2_ (48%), *bla*_NDM-5_ (13%), and *bla*_CMY-42_ (10%) (Table 1).

**TABLE 1:** Combination of extended-spectrum β-lactamase, pAmpC, and carbapenemase enzymes in the 127 Escherichia coli ST410 genomes

Phenotype	Gene	No. of genomes	% of genomes
None	No gene detected	13	10
ESBL	*bla*_CTX-M-15_	21	17
	*bla*_CTX-M-1_	2	2
	*bla*_SHV-12_	1	<1
	*bla*_CTX-M-1_/*bla*_CTX-M-15_	1	<1
pAmpC	*bla*_CMY-42_	7	6
	*bla*_CMY-2_	1	<1
Carbapenemase	*bla*_OXA-181_	1	<1
ESBL/pAmpC	*bla*_CTX-M-15_/*bla*_CMY-2_	1	<1
	*bla*_CTX-M-15_/*bla*_CMY-42_/*bla*_DHA-1_	1	<1
ESBL/carbapenemase	*bla*_CTX-M-15_/*bla*_OXA-181_	3	2
	*bla*_CTX-M-15_/*bla*_KPC-2_	2	2
	*bla*_CTX-M-15_/*bla*_NDM-1_	1	<1
	*bla*_CTX-M-15_/*bla*_KPC-3_	2	2
	*bla*_CTX-M-15_/*bla*_OXA-48_	1	<1
	*bla*_TEM-30_/*bla*_KPC-3_	2	2
pAmpC/carbapenemase	*bla*_CMY-2_/*bla*_OXA-181_	11	9
	*bla*_CMY-2_/*bla*_NDM-5_	1	<1
	*bla*_CMY-42_/*bla*_OXA-181_	1	<1
	*bla*_DHA-1_/*bla*_OXA-48_	1	<1
	*bla*_CMY-2_/*bla*_NDM-5_/*bla*_OXA-181_	2	2
ESBL/pAmpC/carbapenemase	*bla*_CTX-M-15_/*bla*_CMY-2_/*bla*_OXA-181_	29	23
	*bla*_CTX-M-15_/*bla*_CMY-42_/*bla*_OXA-181_	3	2
	*bla*_CTX-M-15_/*bla*_CMY-2_-truncated/*bla*_OXA-181_	2	2
	*bla*_CTX-M-15_/*bla*_CMY-42_/*bla*_OXA-48_	1	<1
	*bla*_CTX-M-15_/*bla*_CMY-2_/*bla*_NDM-5_/*bla*_OXA-181_	9	7
	*bla*_CTX-M-15_/*bla*_CMY-2_/*bla*_NDM-4_/*bla*_OXA-181_	3	2
	*bla*_CTX-M-15_/*bla*_SHV-12_/*bla*_CMY-2_/*bla*_NDM-5_	1	<1
	*bla*_CTX-M-15_/*bla*_SHV-12_/*bla*_CMY-2_/*bla*_NDM-5_/*bla*_OXA-181_	3	2

From the analysis of the quinolone resistance-determining regions (QRDRs) in the 127 ST410 genomes, 115 of the genomes had S83L and D87N amino acid substitutions in *gyrA*, S80I substitution in *parC*, and S458A substitution in *parE*. None of the 127 genomes had amino acid changes in the QRDR of *gyrB* (Table S1).

Two genomes from the United States (SAMN06645838 and SAMN02442857) and one genome from Denmark (SAMN01885884) belonged to *fimH53* (*H53*). The remaining 124 genomes belonged to *fimH24* (*H24*) (Table S1).

Of the 127 ST410 genomes, 66 (52%) had an IncX3 replicon and 125 (98%) had an IncF replicon, with the majority (58/125, 46%) belonging to pMLST F1:A1:B49, while 24 (19%) belonged to F36:A4:B1, seven (6%) belonged to F31:A4:B1, six (5%) belonged to F48:A1:B49, and three (2%) belonged to F2:A4:B1. For the remaining 27 genomes, 23 different pMLSTs were identified (Table S1).

### Epidemiology of E. coli ST410 in Denmark.

**FIG 1:**
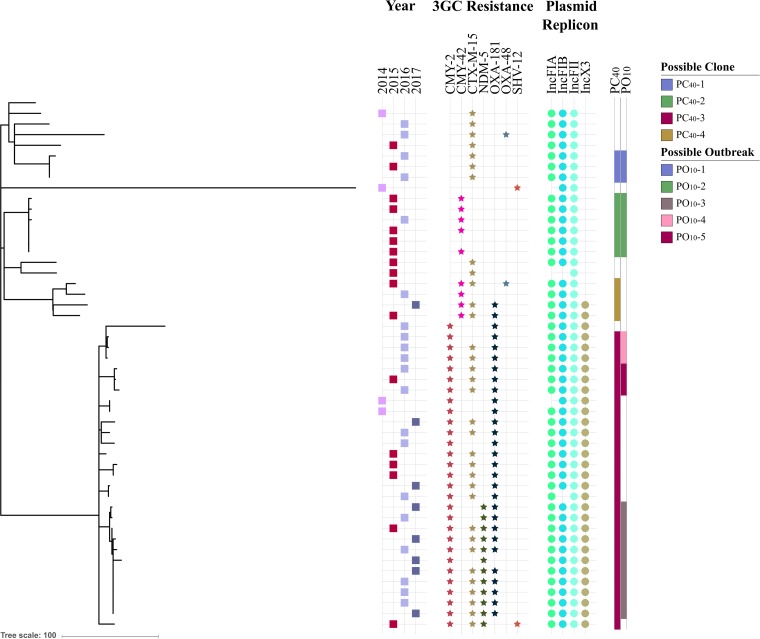
Rooted maximum likelihood phylogeny constructed with Gubbins based on SNP alignment of 49 Danish E. coli ST410 genomes. For each isolate, the following metadata are included: year of isolation, associated third-generation cephalosporin resistance (3GC resistance), and presence of IncFIA, IncFIB, IncFII, and IncX3 replicons. Possible clones and outbreaks are colored as per the key.

The epidemiology of the 49 ST410 isolates from the Danish surveillance program was investigated by single nucleotide polymorphism (SNP) analysis and visualized with the metadata year of isolation, third-generation cephalosporin resistance (3GC-R) genes, and IncX3 and IncF replicons in a phylogenetic tree (Fig. 1). For construction of the phylogenetic tree, 721 SNPs were identified between the 49 genomes, based on 86% of the reference chromosome after removal of recombinant regions.

The SNP analysis was used to identify possible clones (PC) within the ST410 national Danish strain collection. A pairwise comparison of the SNP differences across the isolates (Fig. S1A) showed a group of isolates with no more than 40 SNPs to the nearest neighbor (0 to 41 SNPs in pairwise comparison), two isolates with 80 SNPs between the genomes, and the remainder with more than 150 SNPs to the nearest neighbor. Adhering to an SNP distance of ≤40 SNPs to the nearest neighboring isolate for describing possible clones (PC_40_) (Table 2) resulted in four PC_40_s (PC_40_-1, -2, -3, and -4). Additionally, possible outbreaks (PO; clusters containing at least three individual isolates) were identified by adhering to an SNP distance of ≤10 SNPs to the nearest neighboring isolate (PO_10_). Based on this cutoff value, five possible outbreaks were identified (PO_10_-1, -2, -3, -4, and -5) (Fig. 1).

10.1128/mSphere.00337-18.1 FIG S1  Frequency in percentage of pairwise SNP difference between the 49 ST410 E. coli isolates from Danish patients (A) and between isolates within the possible clones (PC_40_) and the remaining isolates (B). Download FIG S1, TIF file, 0.9 MB. Copyright © 2018 Roer et al. 2018 Roer et al. This content is distributed under the terms of the Creative Commons Attribution 4.0 International license.

**TABLE 2:** Possible clones and outbreaks identified in the Danish ST410 Escherichia coli collection^a^

PC_40_	No. of genomes in PC_40_	Region(s) (no. of genomes)	No. of SNPs	PO_10_	No. of genomes in PO_10_	Region(s) (no. of genomes)	No. of SNPs	Yr(s) (no. of genomes)	Resistance enzyme(s) (no. of genomes)	Replicon(s)
PC_40_-1	3	Ce (3)	0–4	PO_10_-1	3	Ce (3)	0–4	2015 (1), 2016 (2)	CTX-M-15	IncF
PC_40_-2	6	Ce (6)	0–2	PO_10_-2	6	Ce (6)	0–2	2015 (5), 2016 (1)	CMY-2 (5)	IncF
PC_40_-3	28	Ca (13), Ze (10), So (1), Ce (4)	0–41	PO_10_-3	11	Ca (1), Ze (10)	0–12	2015 (1), 2016 (5), 2017 (5)	CMY-2, OXA-181 (10), NDM-5, CTX-M-15 (8)	IncF, IncX3
				PO_10_-4	3	So (1), Ca (2)	3–6	2016 (3)	CMY-2, OXA-181, CTX-M-15 (2)	IncF, IncX3
				PO_10_-5	3	Ca (1), Ce (2)	2–3	2015 (1), 2016 (2)	CMY-2, OXA-181, CTX-M-15	IncF, IncX3
PC_40_-4	4	Ca (4)	15–38							

a Abbreviations: PC_40_, possible clone with ≤40 SNPs to the nearest neighboring isolate; PO_10_, possible outbreak with ≤10 SNPs to the nearest neighboring isolate; Ce, central region of Denmark; Ca, capital region of Denmark; Ze, Zealand region; So, southern region of Denmark.

PC_40_-1 encompassed three genomes with 0 to 4 SNP differences, thus also indicating a possible outbreak (PO_10_-1). The three isolates were from 2015 (*n* = 1) and 2016 (*n* = 2); all three genomes contained the *bla*_CTX-M-15_ gene and an IncF replicon with pMLST F36:A4:B1. Travel information for the three patients was not available, but the patients had all been hospitalized concurrently in the same hospital unit, R-2 (Fig. S3), in the central region of Denmark (map in Fig. S2).

10.1128/mSphere.00337-18.2 FIG S2  Map of Danish regions. Download FIG S2, TIF file, 1.1 MB. Copyright © 2018 Roer et al. 2018 Roer et al. This content is distributed under the terms of the Creative Commons Attribution 4.0 International license.

10.1128/mSphere.00337-18.3 FIG S3  Timeframe and link between patients P1 to P3 in the possible outbreak 1 (PO_10_-1). Admission to a hospital unit shared by other patients (across all five PO_10_s, Fig. S3 to S7) is indicated by colors, where gray indicates admission to hospital units not shared by other patients. The month of isolation of ST410 is indicated by an asterisk. Possible routes of transmission between patients are indicated with black arrows. Download FIG S3, TIF file, 0.3 MB. Copyright © 2018 Roer et al. 2018 Roer et al. This content is distributed under the terms of the Creative Commons Attribution 4.0 International license.

The second possible clone, PC_40_-2, included six genomes with 0 to 2 SNP differences, also indicating a possible outbreak (PO_10_-2). The six isolates were from 2015 (*n* = 5) and 2016 (*n* = 1) and originated from five patients hospitalized in the central region of Denmark. An IncF replicon with pMLST F48:A1:B49 and *bla*_CMY-42_ were present in 5/6 genomes. Travel information was unavailable for all five patients associated with these six isolates. However, patients P1, P3, P4, and P5 were hospitalized concurrently at the same hospital unit (R-8) in the beginning of June, and patients P2 and P4 also simultaneously at R-8 in mid-June (Fig. S4).

10.1128/mSphere.00337-18.4 FIG S4  Timeframe and link between patients P1 to P5 in the possible outbreak 2 (PO_10_-2). Admission to a hospital unit shared by other patients (across all five PO_10_s, Fig. S3 to S7) is indicated by colors, where gray indicates admission to hospital units not shared by other patients. The month of isolation of ST410 is indicated by an asterisk. Possible routes of transmission between patients are indicated with black arrows. Download FIG S4, TIF file, 0.8 MB. Copyright © 2018 Roer et al. 2018 Roer et al. This content is distributed under the terms of the Creative Commons Attribution 4.0 International license.

The third possible clone (PC_40_-3) comprised 28 isolates, collected from patients hospitalized in the capital region of Denmark (*n* = 13), the Zealand region (*n* = 10), the southern region of Denmark (*n* = 1), and the central region of Denmark (*n* = 4). The genomes carried *bla*_CMY-2_ (28/28) and *bla*_OXA-181_ (27/28); IncF replicons with pMLST F1:A1:B49 (25/28), F1:A6:B49 (2/28), or F2:A1:B49 (1/28); and an IncX3 replicon (28/28). PC_40_-3 spanned between 0 and 41 SNPs and featured three possible outbreaks, PO_10_-3, PO_10_-4, and PO_10_-5, described in an epidemiological context below.

PO_10_-3 comprised 11 genomes with 0 to 12 SNP differences, collected from nine patients hospitalized during 2015 (*n* = 1), 2016 (*n* = 5), and 2017 (*n* = 5) in the capital region of Denmark (*n* = 1) and the Zealand region (*n* = 10). For two patients, two isolates were included: patient P3 with 11 SNP differences between the isolates collected 13 months apart and patient P6 with three SNP differences between the isolates collected 5 months apart. The genomes in PO_10_-3 carried *bla*_NDM-5_ (11/11) and *bla*_CTX-M-15_ (8/11), and all genomes harbored IncF (pMLST F1:A1:B49) and IncX3 replicons. A history of travel to Egypt was registered for P1 from 2015, whereas travel history was unavailable for the remaining 10 patients. Patients P2 to P6 and P8 could be linked through overlapping stays within the same hospital units, whereas patient P7 had been hospitalized at the same hospitals units as P2 to P6 and P8 but 2 or more months apart from the other patients (Fig. S5). No link could be established between patient P1 and the remaining patients. For patient P9, no obvious link could be found to the remaining patients, but patient P9 was admitted to hospital H shortly after patient P3, though at different units.

10.1128/mSphere.00337-18.5 FIG S5  Timeframe and link between patients P1 to P9 in the possible outbreak 3 (PO_10_-3). Admission to a hospital unit shared by other patients (across all five PO_10_s, Fig. S3 to S7) is indicated by colors, where gray indicates admission to hospital units not shared by other patients. The month of isolation of ST410 is indicated by an asterisk. Possible routes of transmission between patients are indicated with black arrows. The red arrow indicates a possible hospital link (not hospital unit). Download FIG S5, TIF file, 1.5 MB. Copyright © 2018 Roer et al. 2018 Roer et al. This content is distributed under the terms of the Creative Commons Attribution 4.0 International license.

PO_10_-4 consists of three isolates with 3 to 6 SNP differences, isolated in 2016 (*n* = 3) in the southern region of Denmark (*n* = 1) and the capital region of Denmark (*n* = 2). The genomes carried *bla*_CTX-M-15_ (2/3), and all harbored IncF (pMLST F1:A1:B49) and IncX replicons. The three patients did not travel prior to hospitalization. Patients P1 and P3 had been hospitalized 4 months apart in the same hospital unit, but no link could be found to patient P2 (Fig. S6).

10.1128/mSphere.00337-18.6 FIG S6  Timeframe and link between patients P1 to P3 in the possible outbreak 4 (PO_10_-4). Admission to a hospital unit shared by other patients (across all five PO_10_s, Fig. S3 to S7) is indicated by colors, where gray indicates admission to hospital units not shared by other patients. The month of isolation of ST410 is indicated by an asterisk. Possible routes of transmission between patients are indicated with black arrows. For PO_10_-4, no link between patients is found. Download FIG S6, TIF file, 0.4 MB. Copyright © 2018 Roer et al. 2018 Roer et al. This content is distributed under the terms of the Creative Commons Attribution 4.0 International license.

PO_10_-5 consisted of three isolates with 2 to 3 SNP differences; they were isolated in 2015 (*n* = 1) and 2016 (*n* = 2), in the capital region of Denmark (*n* = 1) and the central region of Denmark (*n* = 2). The genomes contained *bla*_CTX-M-15_ (3/3), and all harbored IncF (pMLST F1:A1:B49) and IncX3 replicons. A history of travel to Lebanon was registered for P1 from 2015, while for the remaining two patients, one had no history of recent travel and for the other information on travel history was unavailable. Patients P1 and P3 were admitted at different hospitals, whereas patient P2 was not hospitalized within the investigated time period (Fig. S7).

10.1128/mSphere.00337-18.7 FIG S7  Timeframe and link between patients P1 to P3 in the possible outbreak 5 (PO_10_-5). Admission to a hospital unit shared by other patients (across all five PO_10_s, Fig. S3 to S7) is indicated by colors, where gray indicates admission to hospital units not shared by other patients. The month of isolation of ST410 is indicated by an asterisk. Possible routes of transmission between patients are indicated with black arrows. For PO_10_-5, no link between patients is found. Download FIG S7, TIF file, 0.2 MB. Copyright © 2018 Roer et al. 2018 Roer et al. This content is distributed under the terms of the Creative Commons Attribution 4.0 International license.

The fourth possible clone (PC_40_-4) included four isolates with 15 to 38 SNP differences. As the pairwise comparison showed >10 SNPs for all combinations, no regional outbreaks were inferred for this clone. All four isolates carried an IncF replicon with various pMLST profiles (F36:A4:B1, 2/4; F31:A4:B1, 1/4; or F2:A6:B33, 1/4), and two of the isolates carried an IncX3 replicon. The isolates were from 2015 (*n* = 2), 2016 (*n* = 1), and 2017 (*n* = 1) from patients hospitalized in the capital region of Denmark (*n* = 4). Two of the patients had a history of travel to Pakistan, while travel history was unavailable for the remaining two patients.

### E. coli ST410 in a global context.

The 127 ST410 international isolates spanned more than 4 decades (1975 to 2017) and were from 14 different countries: Denmark (*n* = 51); United Kingdom (*n* = 32); United States (*n* = 20); Germany (*n* = 10); Canada (*n* = 4); Brazil (*n* = 2); and Ireland, Japan, Nepal, Norway, Saudi Arabia, Singapore, Sweden, and Tanzania (one isolate each).

**FIG 2:**
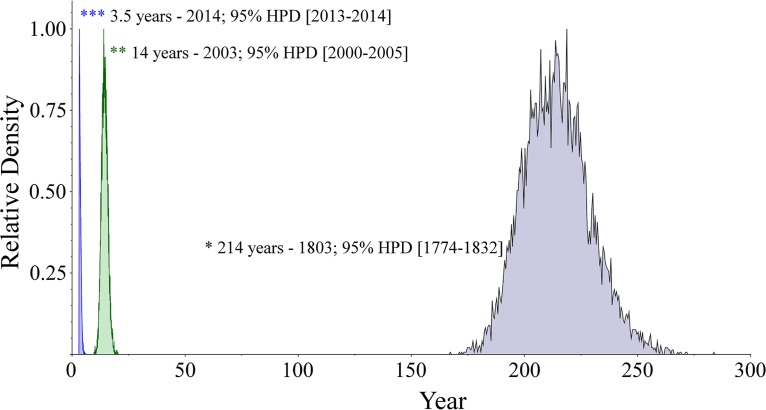
Introduction of OXA-181 and NDM-5 into E. coli ST410. Peaks in the density diagram are colored by the introduction of the carbapenemases: green, OXA-181; blue, NDM-5.

**FIG 3:**
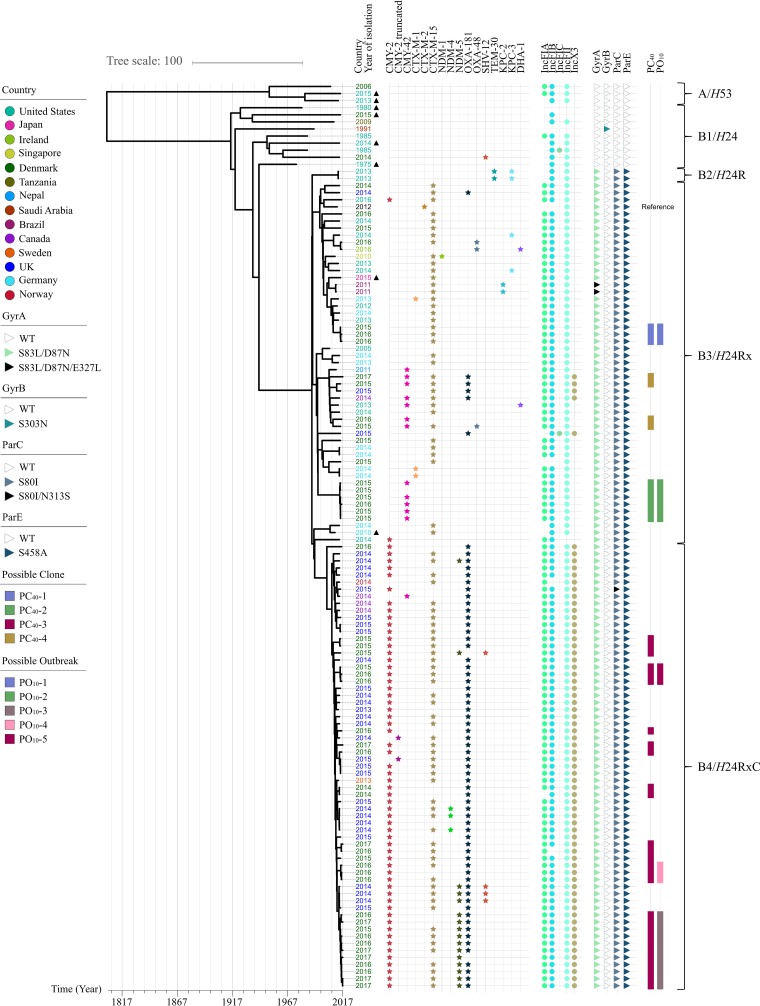
Time-scaled phylogeny of E. coli ST410 (*n* = 127) with associated third-generation cephalosporin resistance; plasmid replicons; mutations in the QRDR of *gyrA*, *gyrB*, *parC*, and *parE*; and the possible Danish outbreaks as colored by key. Brackets at right represent ST410 clades as described in the text. The tips of the tree to the left represent the year of isolation for the isolates, with the year stated next to the tips, which is colored by country of isolation, as per the key. ▲, genomes isolated from sources other than human. WT, wild type.

To investigate ST410 in a global context and its temporal dissemination and evolution, a Bayesian coalescent method was applied for phylogenetic reconstruction of the ST410 lineage based on the genomic data and sampling time. By using a general time-reversible (GTR) model and a strict molecular clock with a Bayesian Skyline population model, the BEAST analysis estimated the age of the ST410 lineage to be approximately 214 years (1803; 95% highest posterior density [HPD], 1774 to 1832) (Fig. 2). The phylogenetic reconstruction revealed two distinct clonal lineages of ST410: lineage A with *fimH53* (A/H53) and lineage B with *fimH24* (B/H24) (Fig. 3). The B/H24 lineage was further divided into three sublineages: B2/H24R with the introduction of fluoroquinolone resistance by mutations in *gyrA* and *parC*, B3/H24Rx with the introduction of *bla*_CTX-M-15_, and B4/H24RxC with the introduction of *bla*_OXA-181_.

PC_40_-1, PC_40_-2, and PC_40_-4 were all part of the B3/H24Rx complex (Fig. 3). The Danish ST410 isolates in PC_40_-1 and PC_40_-2 from the national analysis did not cluster together with any non-Danish isolates (Fig. 3). The four Danish isolates from PC_40_-4 clustered together with one isolate from the United Kingdom, one isolate from Canada, and two isolates from the United States. For PC_40_-3, an additional 35 isolates clustered together with the 28 Danish isolates, resulting in a distinct clade with 63 isolates (B4/H24RxC in Fig. 3).

PO_10_-4 and PO_10_-5 clustered in separate clades without any additional isolates, whereas PO_10_-3 clustered closely with four isolates from the United Kingdom (7 to 21 SNPs to the nearest neighbor). These 63 isolates were collected between 2013 and 2017, in Denmark (*n* = 29), the United Kingdom (*n* = 29), Canada (*n* = 3), Norway (*n* = 1), and Sweden (*n* = 1). Additionally, 47 of the isolates in B4/H24RxC carried *bla*_OXA-181_, two isolates carried *bla*_NDM-5_, and 14 isolates carried both carbapenemases.

### Introduction of *bla*_OXA-181_ and *bla*_NDM-5_ into ST410.

The timing of introductions of *bla*_OXA-181_ and *bla*_NDM-5_ into B4/H24RxC was predicted using BEAST (Fig. 3). The introduction of *bla*_OXA-181_ was estimated to have occurred approximately 14 years ago (2003; 95% HPD, 2000 to 2005), with the subsequent introduction of *bla*_NDM-5_ 3.5 years ago (2014; 95% HPD, 2013 to 2014).

To investigate if *bla*_OXA-181_ was carried on an IncX3 plasmid, the genome scaffolds of the 63 ST410 genomes in B4/H24RxC with *bla*_OXA-181_ and/or IncX3 and an additional six genomes with *bla*_OXA-181_ and/or IncX3 were compared with the complete plasmid sequence of pAMA1167-OXA-181 from E. coli ST410 (10) (Fig. S8A). Overall, the results indicated that the plasmid harboring *bla*_OXA-181_ is well preserved; only one *bla*_OXA-181_-positive ST410 isolate, from the United Kingdom, did not carry the IncX3 plasmid (the outermost ring, H14352020701). Additionally, two isolates from Denmark harbored the IncX3 backbone but did not carry the *bla*_OXA-181_ gene (CPO20150034 and CPO20170049).

10.1128/mSphere.00337-18.8 FIG S8  OXA-181- and/or IncX3-positive genomes were compared with the pAMA1167-OXA-181 plasmid (A), and the NDM-5- and/or IncF[F1:A1:B49]-positive genomes were compared with the pAMA1167-NDM-5 plasmid (B), by using the GView server. The concentric rings display hits with minimum 80% similarity between the reference plasmids in the inner ring and the genome scaffolds. The arrows indicate resistance genes and plasmid replicons of the reference plasmids, according to the orientation on the DNA strand. The rings of the genome scaffolds are ordered as in Fig. 3 and colored by country as per the key in Fig. 3. Download FIG S8, TIF file, 2.6 MB. Copyright © 2018 Roer et al. 2018 Roer et al. This content is distributed under the terms of the Creative Commons Attribution 4.0 International license.

To investigate if *bla*_NDM-5_ was introduced via an IncF plasmid with pMLST F1:A1:B49, the 58 ST410 draft genomes with *bla*_NDM-5_ and/or IncF with pMLST F1:A1:B49 were compared with the complete plasmid sequence of pAMA1167-NDM-5 (11) (Fig. S8B). All 58 isolates carried genomic information, which covered major parts of the pAMA1167-NDM-5 plasmid backbone, regardless of their *bla*_NDM-5_ gene status. However, among the 16 *bla*_NDM-5_-positive genomes, the IncF backbone revealed even higher similarity. The 11 Danish ST410 isolates from PO_10_-3, and four United Kingdom isolates clustering near the PO_10_-3 group, all carried similar IncF backbones (15 innermost rings in Fig. S8B [see also Fig. S9]). However, some of the plasmids lacked various resistance genes, including one United Kingdom isolate (ring 12 from the middle, H15074073205) missing the *bla*_NDM-5_ gene. Finally, three of the United Kingdom isolates carried *bla*_NDM-4_ (from the middle; ring 22, H14148045301; 24, H14142058201; and 25, H14140075501) on a plasmid very similar to the *bla*_NDM-5_ plasmid found in the PO_10_-3 isolates.

10.1128/mSphere.00337-18.9 FIG S9  Fifteen innermost rings of Fig. S8B. BLAST of *bla*_NDM-5_- and/or IncF[F1:A1:B49]-positive genomes against pAMA1167-NDM-5 by using the GView server. The concentric rings display similarity between the reference plasmids in the inner ring and the genome scaffolds. The arrows indicate resistance genes and plasmid replicons of the reference plasmids, according to the orientation on the DNA strand. The rings of the genome scaffolds are colored by country as per the key in Fig. 3. Download FIG S9, TIF file, 2 MB. Copyright © 2018 Roer et al. 2018 Roer et al. This content is distributed under the terms of the Creative Commons Attribution 4.0 International license.

## DISCUSSION

Recent studies indicate that E. coli ST410 is another successful pandemic extraintestinal pathogenic *E. coli* (ExPEC) lineage similar to ST131 (5, 16). Our findings support this hypothesis; however, as national surveillance programs monitor only local epidemiology, global surveillance is required to follow the dissemination of pandemic clones. The limited number of studies on how to interpret phylogenetic relatedness based on SNP differences in E. coli makes it difficult to give a generic threshold on how to distinguish clones from local or regional outbreaks when epidemiological data are not available (17–19). Accessing the national collection of E. coli ST410 from Denmark, a cutoff value of no more than 40 SNP differences to the nearest neighbor seemed to define clones within this collection, as the closest genome differed by 159 to 185 SNPs to PC_40_-1 to -4 (see Fig. S1B in the supplemental material). The definition of outbreaks being 10 or fewer SNP differences apart from the nearest neighbor has previously been shown plausible (9) and was thus used in this study. Breakdown into possible outbreaks (≤10 SNPs) revealed five possible ST410 outbreaks (PO_10_s) in Denmark during 2015 to mid-2017. Two of these, PO_10_-1 and PO_10_-2, carried *bla*_CTX-M-15_ and *bla*_CMY-42_, respectively. Travel history for all patients in these two PO_10_s was unavailable, and comparison with the genomes collected from EnteroBase did not reveal any links to other countries. Isolates within these two PO_10_s were collected from patients within the same region. Epidemiological investigation revealed links between the patients indicating that both could be true outbreaks. This is further supported by the global analysis, where the Danish outbreaks cluster in distinct clades (Fig. 3).

The possible clone PC_40_-3, including PO_10_-3, -4, and -5, consisted of 28 isolates with 0 to 41 SNP differences, and most of the isolates carried *bla*_CTX-M-15_, *bla*_CMY-2_, and *bla*_OXA-181_. Regarding PO_10_-4 and PO_10_-5, no epidemiological link could be made between the patients within the possible outbreaks. In PO_10_-4, the patients did not travel prior to hospitalization, and in PO_10_-5, one patient had recent travel activity to Lebanon, one patient did not report travel, and travel history was unavailable for the last patient. Thus, it is unclear if multiple introductions occurred, or if unknown carriers or contaminated environmental sources could link the spread between patients from these two PO_10_s.

Further analysis revealed another possible outbreak (PO_10_-3) involving 11 isolates from nine patients (P1 to P9), where one patient (P6) contributed with two samples isolated 5 months apart and one patient (P3) contributed with two samples 13 months apart. Isolates from PO_10_-3 had an additional carbapenemase gene, *bla*_NDM-5_. An epidemiological link was found among patients P2 to P8. The patient P9 could possibly be linked to this outbreak via hospital H. We were unable to link patient P1 to the other patients. However, it is not possible to exclude an unknown carrier of the clone, nor an unregistered travel link for patients P2 to P9, whereas patient P1, with the isolate from 2015 (AMA1167), had traveled to Egypt prior to isolation of AMA1167 (10). Also in 2015, an E. coli ST410 isolate harboring *bla*_CTX-M-15_ and *bla*_CMY-2_ and carrying *bla*_OXA-181_ on an ~48.5-kb plasmid and *bla*_NDM-5_ on an ~100-kb plasmid together with FIA and FIB replicons was reported in a study from Egypt (20). The characteristics of this Egyptian OXA-181/NDM-5-producing ST410 isolate correlate with the first OXA-181/NDM-5-producing ST410 isolate collected in Denmark, suggesting that the Danish patient, P1, most likely acquired the isolate during travel to Egypt in 2015. Unfortunately, no WGS data are available for the Egyptian isolate to evaluate such a link.

From the global analysis, the close genetic relationship between four ST410 isolates isolated in the United Kingdom and the genomes from Danish ST410 isolates from PO_10_-3 could indicate a similar introduction to the United Kingdom; however, two of the patients from the United Kingdom reported no travel prior to hospitalization, and for the remaining two, travel information was unavailable. Similar genetic profiles have previously been reported for NDM-producing E. coli ST410 isolates obtained from patients in Denmark and the United Kingdom (21), indicating either similar travel patterns by inhabitants of the two countries or direct transmission between the countries. However, comprehensive epidemiological data with travel information are needed on a global scale to clarify the actual route of transmission.

The coalescence analysis (Fig. 2 and 3) on the global collection of 127 ST410 isolates indicated a most recent common ancestor approximately 214 years ago (the early 1800s), with confidence intervals stretching 30 years backward and forward. In comparison, the time to the most recent common ancestor for the ST131 clade was estimated as the late 1800s (22). Therefore, compared with ST131, ST410 is predicted to be a slightly older lineage of E. coli, even though the prevalence of ST131 is far higher than that of ST410.

Several studies have investigated the population structure of ST131 with WGS data and found a sublineage, C/H30, defined by the presence of the *fimH30* allele. Within the C/H30 lineage, the C1/H30R clade, possessing fluoroquinolone resistance conferred by mutations in the chromosomal genes *gyrA* and *parC*, and the C2/H30Rx clade, which in addition is associated with carriage of *bla*_CTX-M-15_, emerged around 1987 (3, 4, 22, 23). We identified a similar population structure of ST410, with two sublineages based on the *fimH* alleles: A/H53 (3/127 genomes) and B/H24 (124/127 genomes). In the B/H24 sublineage, the introduction of the fluoroquinolone resistance by mutations in *gyrA* and *parC* (B2/H24R) and the introduction of *bla*_CTX-M-15_ (B3/H24Rx) were also estimated to have occurred around 1987 concurrently with the widespread clinical introduction of extended-spectrum cephalosporins and fluoroquinolones (Fig. 3). Additionally, the introduction of *bla*_CTX-M-15_ seems to be related to the acquisition and persistence of an IncFII plasmid harboring FIA/FIB replicons, which was also observed in ST131 (22). In ST410, the introduction of *bla*_OXA-181_ on an IncX3 plasmid was predicted to have occurred around 2003 (Fig. 3). Furthermore, introduction of *bla*_NDM‑5_, likely on an IncF replicon, around 3.5 years ago, resulted in a highly resistant clone causing an outbreak (PO_10_-3) in Denmark.

The majority of the 127 international ST410 isolates in our study were collected from humans (119/127); however, eight isolates were from nonhuman sources. The genomes obtained from isolates from swine (*n* = 2), poultry (*n* = 2), and air from poultry houses (*n* = 2) were all located in the nonresistant clades A/H53 and B1/H24 (Fig. 3), whereas the genomes obtained from sewage and from a dog were both located in the B3/H24Rx cluster. The latter cases likely represent the shared environment with humans. In our study, isolates found in the carbapenemase clade B4/H24RxC were all of human origin. However, carbapenemase genes are often located on mobile genetic elements (13, 14), together with other resistance genes encoding resistance to antibiotic classes commonly used in veterinary medicine (e.g., tetracycline, sulfonamides, or phenicols). Thus, once introduced in animals, carbapenemase genes may be coselected by the use of other classes of antibiotics, which could increase the prevalence of CPE in animals.

CPE is a worrisome risk in public health due to the multidrug resistance and the potential for dissemination to multiple sources, including transmission from patient to patient. The mobile colistin resistance gene *mcr-1* has been reported in ESBL-producing E. coli ST410 in Brazil (24) and Germany (25) but was not observed in our 127 isolates. This demonstrates the potential of ST410 to acquire further resistance determinants, leaving very limited therapeutic agents for treating life-threatening bacterial infections.

ST410 E. coli represent a globally distributed lineage isolated in Europe, North America, South America, Asia, and Africa and associated with various antimicrobial resistance determinants, including ESBLs, pAmpCs, carbapenemases, and colistin resistance genes. Moreover, our data indicate that ST410 has the ability to persist within a host for long intervals. A subsequent sampling of two patients resulted in the detection of two ST410 isolates in both samples, the first patient with 11 SNP differences between the genomes of isolates collected 13 months apart and the second patient with three SNP differences between the genomes of isolates collected 5 months apart.

The possible outbreaks presented here and previous observations of interspecies transmission of ST410 (5) indicate a potential for effective transmission between patients. Additionally, among the Danish ST410 isolates, 20/49 (41%) were from bloodstream infections, which may qualify for severe infections, and show a potential for enhanced pathogenicity. ST410 belongs to phylogroup A, generally linked to commensal colonization (26), whereas ST131 E. coli belongs to phylogroup B2, dominated by strains associated with extraintestinal human infections, adherent-invasive E. coli (AIEC), and enteropathogenic E. coli (EPEC) (27). Our study demonstrates that parallels can be drawn between the phylogeny and emergence of fluoroquinolone and ESBL resistance (CTX-M-15) in ST131 and ST410. The reduced virulence coupled to phylogroup A could explain why ST410 has not been as globally dominant as ST131, even though ST410 is a slightly older lineage. Further studies are needed to give the complete and true picture of the pathogenicity and fitness of ST410.

These characteristics are properties of an international high-risk clone (1) and underline that ST410 qualifies as a lineage with new international multidrug-resistant high-risk clones, which should be monitored closely in the future.

## MATERIALS AND METHODS

### Bacterial isolates.

Initially, the strain collection at Statens Serum Institut (SSI) containing all ESBL/pAmpC-producing E. coli isolates from human bloodstream infections and the carbapenemase-producing organisms (CPO) collected by the Danish Departments of Clinical Microbiology (DCMs) as part of the Danish surveillance program DANMAP between 2014 and mid-2017 were screened for E. coli ST410. From the ESBL/pAmpC collection, 14 ST410 isolates from 2014 to 2015 published by Roer et al. (9) and six ST410 isolates from 2016 were obtained (in total, covering 20 isolates from 19 patients). From the CPO surveillance from January 2014 to July 2017, 29 ST410 isolates were included from 27 patients, resulting in a total study population of 49 ST410 isolates from Danish patients. Reported travel information was obtained from the corresponding DCMs as follows: travel-associated and country information (if known), no recent travel activity, or information not available.

Additionally, EnteroBase (https://enterobase.warwick.ac.uk/) was searched for E. coli ST410 genomes (accessed 3 July 2017) to extend the study with internationally available ST410 genomes to estimate when the two carbapenemase genes *bla*_OXA-181_ and *bla*_NDM-5_ were introduced into the ST410 lineage. Only ST410 genomic data with relevant metadata (year of collection, country, and source type) and availability of raw reads were included. The selection criteria resulted in 43 international E. coli genomes: 36 reported to have been collected from humans, two collected from swine, two collected from poultry, two air samples from poultry houses, and one from sewage (see Table S1 in the supplemental material). Finally, sequences from other national surveillance programs with available genome data were included in the study to expand the number and diversity of ST410 genomes harboring *bla*_OXA-181_ with or without *bla*_NDM-5_. Raw reads from 32 genomes were included from the United Kingdom of human origin, one genome from Germany collected from a dog, and one genome each from Norway and from Sweden, both of human origin, resulting in 127 ST410 genomes with relevant metadata and raw reads available for further analysis (Table S1).

### Whole-genome sequencing.

For isolates that were part of DANMAP surveillance, genomic DNA was extracted (DNeasy Blood and Tissue kit; Qiagen, Copenhagen, Denmark), with subsequent library construction (Nextera kit; Illumina, Little Chesterford, United Kingdom) and finally WGS (MiSeq or NextSeq; Illumina) according to the manufacturer’s instructions to obtain paired-end reads of 2 by 250 or 2 by 150 bp in length.

The raw reads of all 127 isolates were assembled into draft genomes using the SPAdes assembler v. 3.10.1 (28).

### Genotypic characterization.

Draft genomes for the 127 ST410 isolates were submitted to the Center for Genomic Epidemiology (CGE) server through the batch uploader developed for the Bacterial Analysis Platform (BAP) (https://cge.cbs.dtu.dk/services/cge/) (29) and analyzed using the CGE tools described below (analysis performed between 14 August 2017 and 24 August 2017).

### (i) Resistance genes and mutations in the QRDRs.

Resistance genes were identified with ResFinder 2.1 (30) (included in the CGE BAP), using a threshold of 100% identity (ID) for identifying genes encoding β-lactamases and carbapenemases and 98.00% ID for all other genes encoding transferable antimicrobial resistance. For phenotypically resistant isolates without β-lactamase- and carbapenemase-encoding genes or isolates with less than 100% identity, KmerResistance 1.0 (https://cge.cbs.dtu.dk/services/KmerFinder/) (31) was used to confirm the presence/absence of the gene(s).

To identify mutations in the QRDRs, a nucleotide BLAST database of the 127 ST410 genomes was set up using BLAST 2.6.0+ (32). Gene sequences of *gyrA*, *gyrB*, *parC*, and *parE* were extracted from all genomes by performing a BLASTN query of a representative nucleotide sequence for each of the four genes against this database. The nucleotide sequences were translated into corresponding amino acid sequences based on standard genetic code, using the reading frame beginning from the start codon. Amino acid changes were identified in the QRDRs (GyrA, amino acid position 67 to 106 [33]; GyrB, amino acid position 331 to 480 [34]; ParC, amino acid position 38 to 169 [35]; and ParE, amino acid position 365 to 525 [36]) for each gene by comparison with the wild-type gene variants of the quinolone- and fluoroquinolone-susceptible E. coli K-12 MG1655 (GenBank accession no. NC_000913.3).

### (ii) Typing of isolates.

Multilocus sequence type (MLST) was extracted with MLST 1.6 (37), as part of the CGE BAP, to verify that the genomes belonged to ST410 in the Achtman scheme (MLST 1) (38).

*fimH* subtypes were identified with FimTyper 1.0 (https://cge.cbs.dtu.dk/services/FimTyper/) (39).

### (iii) Plasmid analysis.

IncF and IncX3 plasmid replicons were identified using PlasmidFinder 1.4 (40) (included in CGE BAP) and further subtyped by plasmid MLST (pMLST) with the pMLST 1.4 tool (40).

The genome of the Danish ST410 isolate AMA1167 has previously been completely sequenced (GenBank accession no. GCA_002803905.1) (10), and the complete sequences of the OXA-181 and NDM-5 plasmids were used for comparative plasmid analysis. BLAST analysis of draft genome against pAMA1167-OXA-181 (GenBank accession no. CP024806.1) and pAMA1167-NDM-5 (GenBank accession no. CP024805.1) was performed using the GView server (https://server.gview.ca/).

### Genomic epidemiology. (i) Single nucleotide polymorphism analysis.

The Northern Arizona SNP Pipeline (NASP) v1.0.0 was used to estimate the molecular epidemiology by identifying SNPs. Briefly, duplicate regions of the reference chromosome of ST410 isolate YD786 (GenBank accession no. NZ_CP013112.1) were identified by aligning the reference against itself with NUCmer v3.1 and masked from downstream analysis. Raw reads of the analyzed genomes were aligned against the FASTA-formatted reference using the Burrows-Wheeler Aligner (BWA), and SNPs were identified using GATK UnifiedGenotyper (41).

Gubbins v2.3.1 was used to remove recombination signals in the SNP output (42). The SNP phylogenies were annotated with relevant metadata using iTOL (http://itol.embl.de) (43).

### (ii) Identification of possible clones, outbreaks, and patient links.

Possible clones (PC) in the Danish collection were predicted based on the distribution of the pairwise SNP differences between the isolates. Possible clonal outbreaks were identified for clusters containing at least three individual isolates, by adhering to an SNP distance of ≤10 SNPs to the nearest neighboring isolate (PO_10_), as previously described (9).

Patient links in the different possible outbreaks (PO_10_s) were investigated 6 months prior to the first identified isolate in the respective PO_10_ and 6 months after the last identified isolate. Hospital admissions (hospital, unit, and time of admission) were used to construct the timeframe and possible routes of transmission for the PO_10_s.

### (iii) Coalescence-based analyses.

BEAST analysis was conducted to reconstruct the global phylogeny of ST410 based on the SNP data generated from the 127 raw sequence data sets, for estimation of the time points wherein *bla*_OXA-181_ and *bla*_NDM-5_ were acquired by the ST410 lineage. For defining the two groups for *bla*_OXA-181_ and *bla*_NDM-5_, a maximum likelihood phylogeny was estimated with PhyML 3.0 by using the Bayesian information criterion with BIONJ as initial tree, nearest neighbor interchange (NNI) as type of tree improvement, and a bootstrap value of 100 (44). Based on the phylogeny, the two groups were defined.

The timing of the acquisitions of *bla*_OXA-181_ and *bla*_NDM-5_ was inferred using coalescence-based analyses in BEAST 1.8.4 at CIPRES Science Gateway (https://www.phylo.org) using duplicate runs. Analyses were performed using different substitution models (general time-reversible [GTR] and Hasegawa-Kishino-Yano [HKY]), strict and relaxed molecular clock, and different demographic models including Bayesian Skyline, constant population, and exponentially growing population tree priors. Chains were sampled every 40,000th step during the 400 million Markov chain Monte Carlo (MCMC) steps. The first 10% of each chain was discarded as burn-in. The best model was selected based on Bayes factor (BF) comparison (45). Data were analyzed with Tracer v1.5 and TreeAnnotator v1.8.4, and the trees were depicted by using iTOL (http://itol.embl.de/) (43).
