# Supplementary figures and images for: Escherichia coli Sequence Type 410 Is Causing New International High-Risk Clones
Source: mSphere. 2018 Jul 18;3(4):e00337-18. doi: 10.1128/mSphere.00337-18 (PMC6052333; doi:10.1128/mSphere.00337-18)

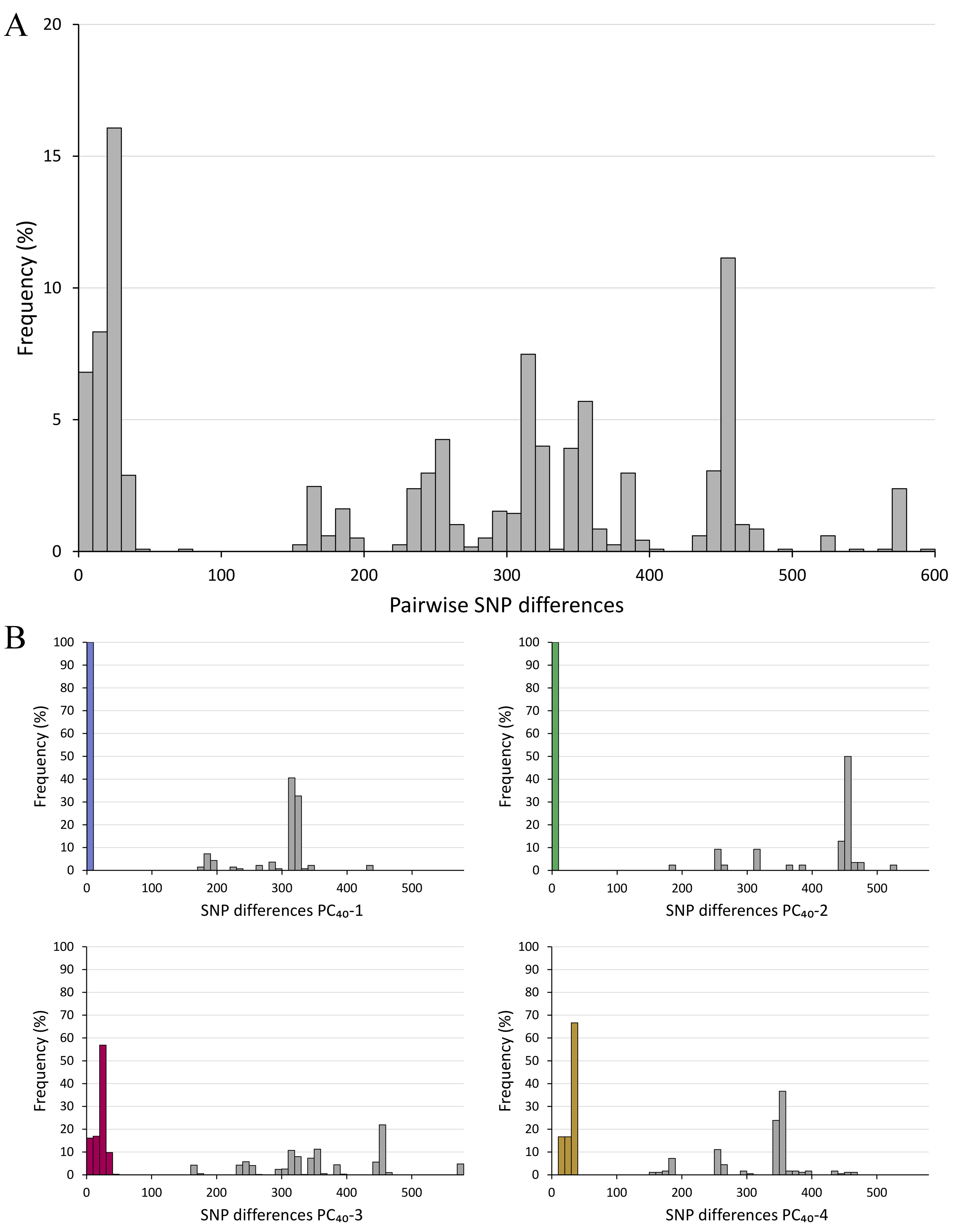

Supplement: FIG S1 [file sph004182593sf1.tif]

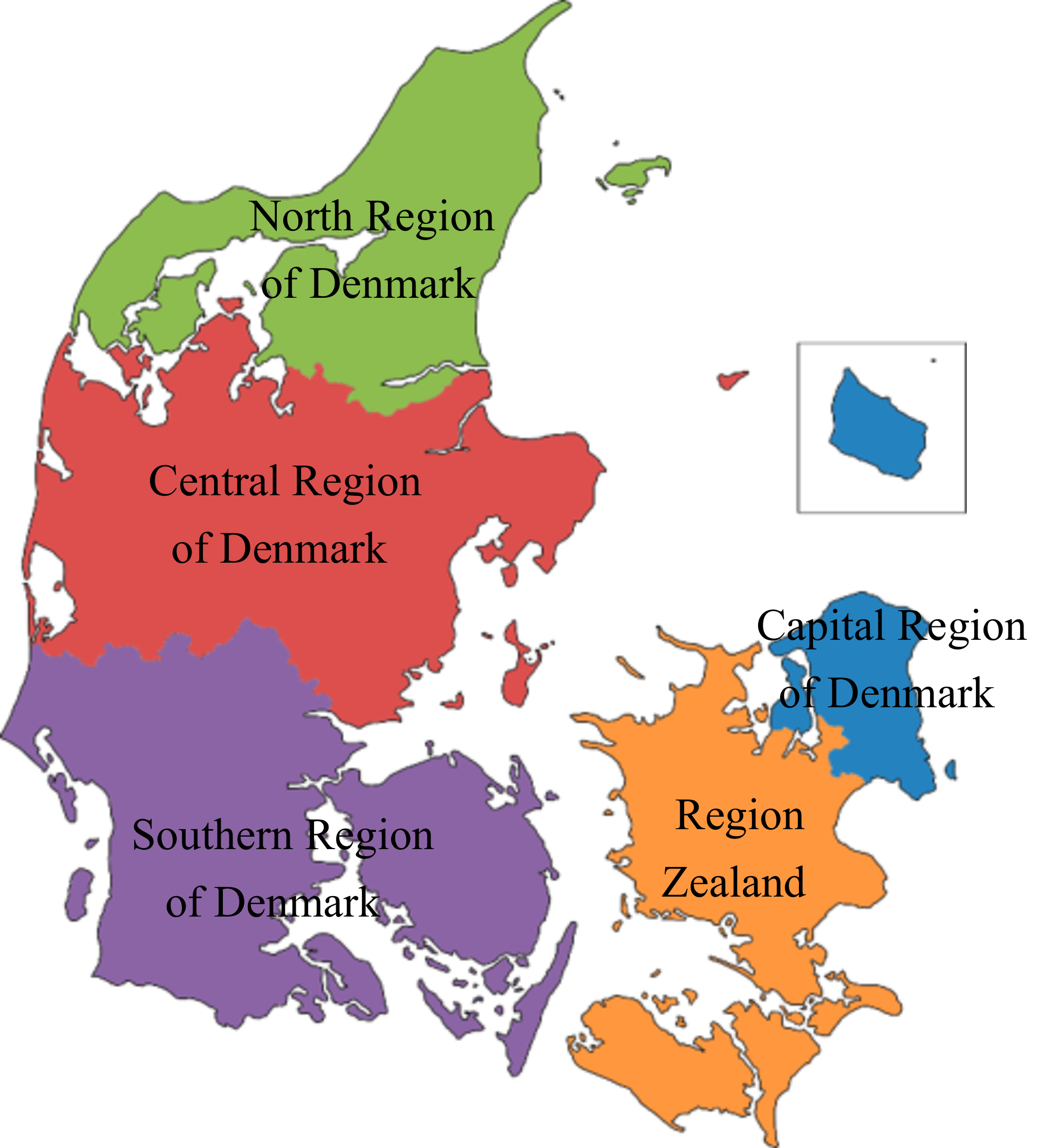

Supplement: FIG S2 [file sph004182593sf2.tif]

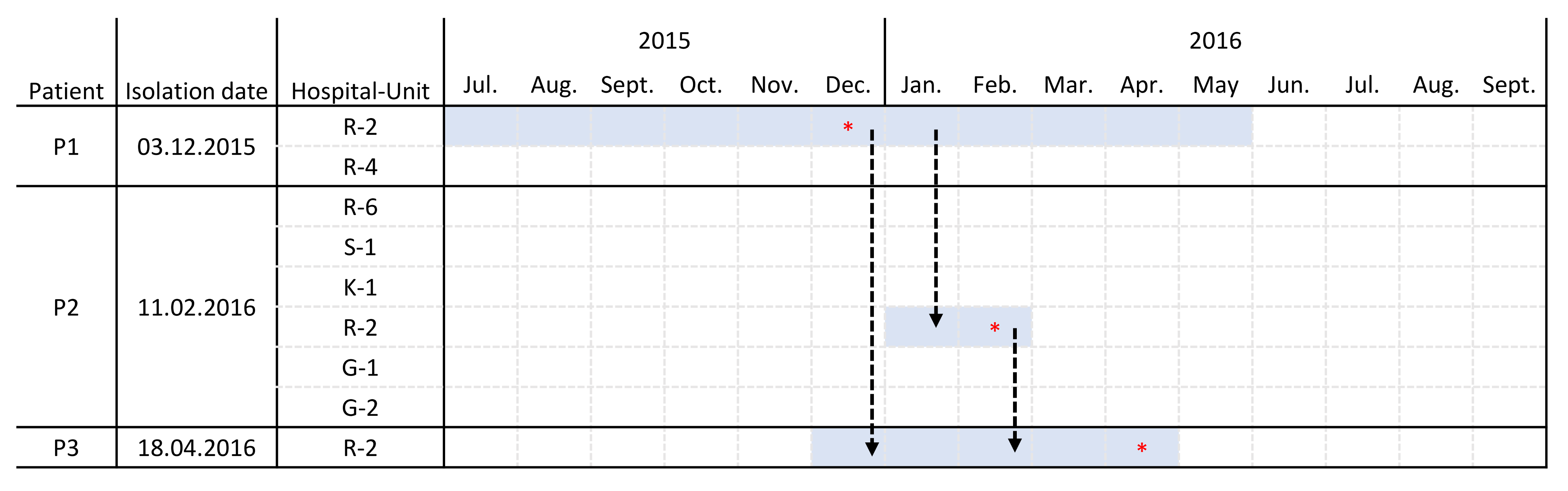

Supplement: FIG S3 [file sph004182593sf3.tif]

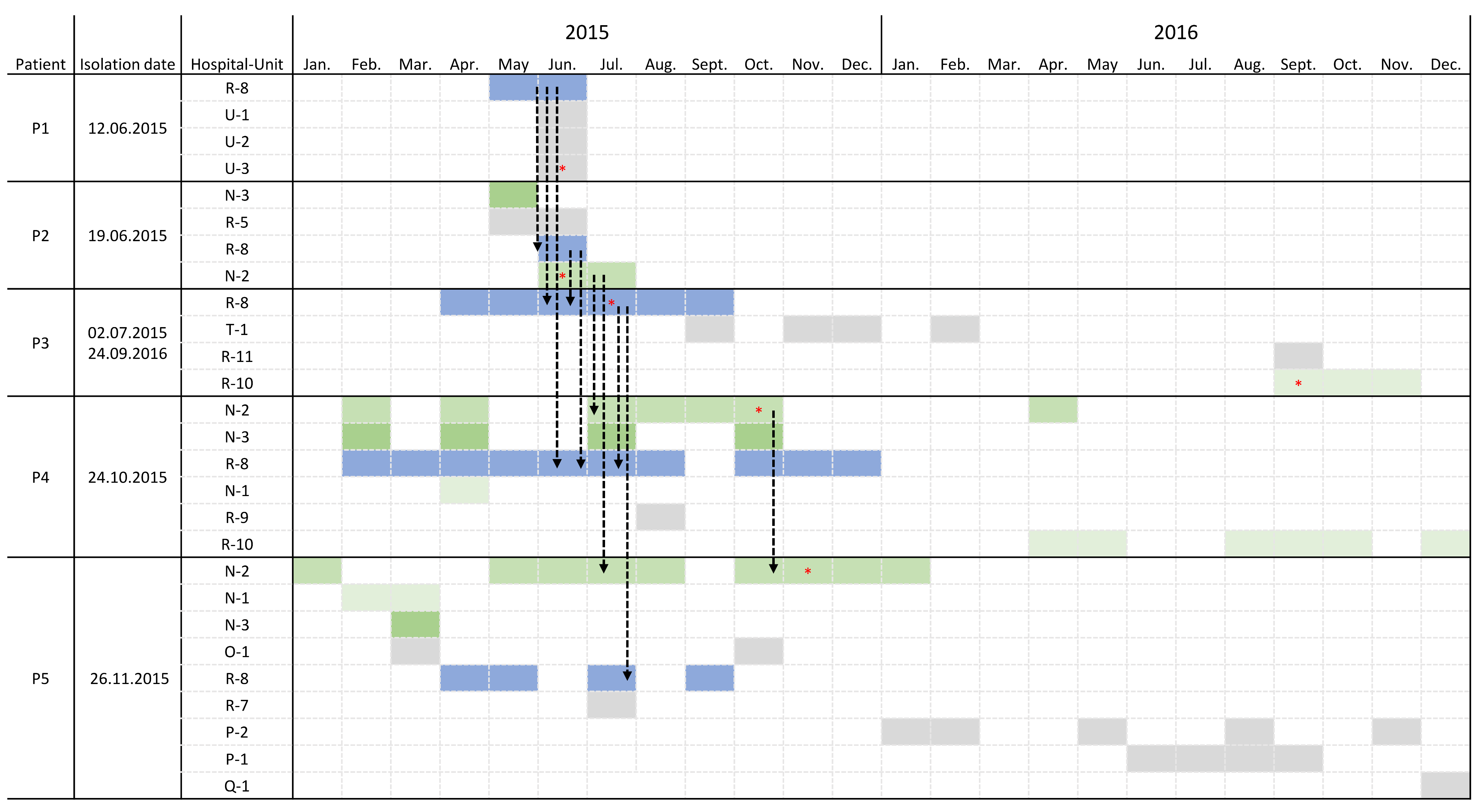

Supplement: FIG S4 [file sph004182593sf4.tif]

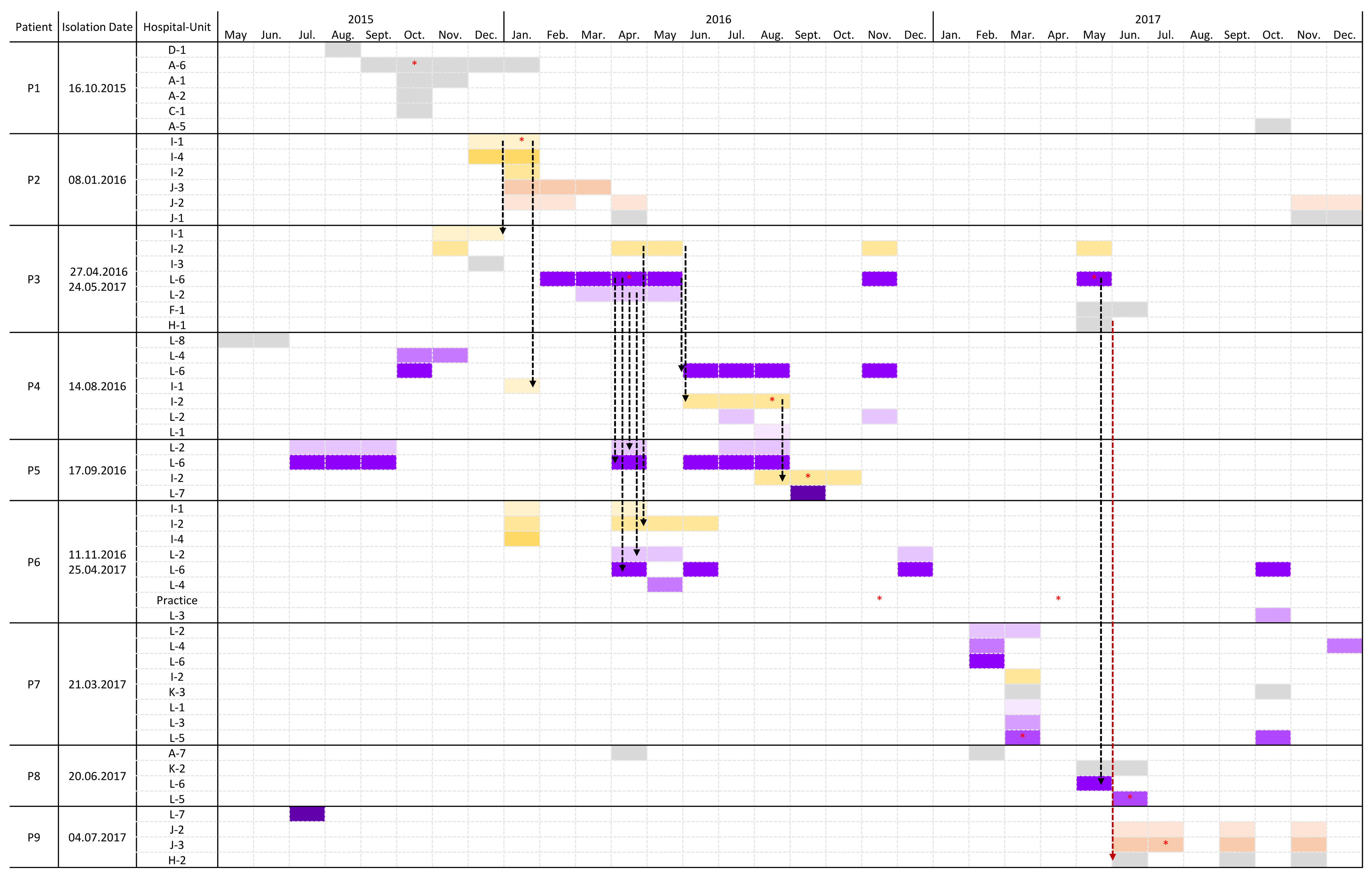

Supplement: FIG S5 [file sph004182593sf5.tif]

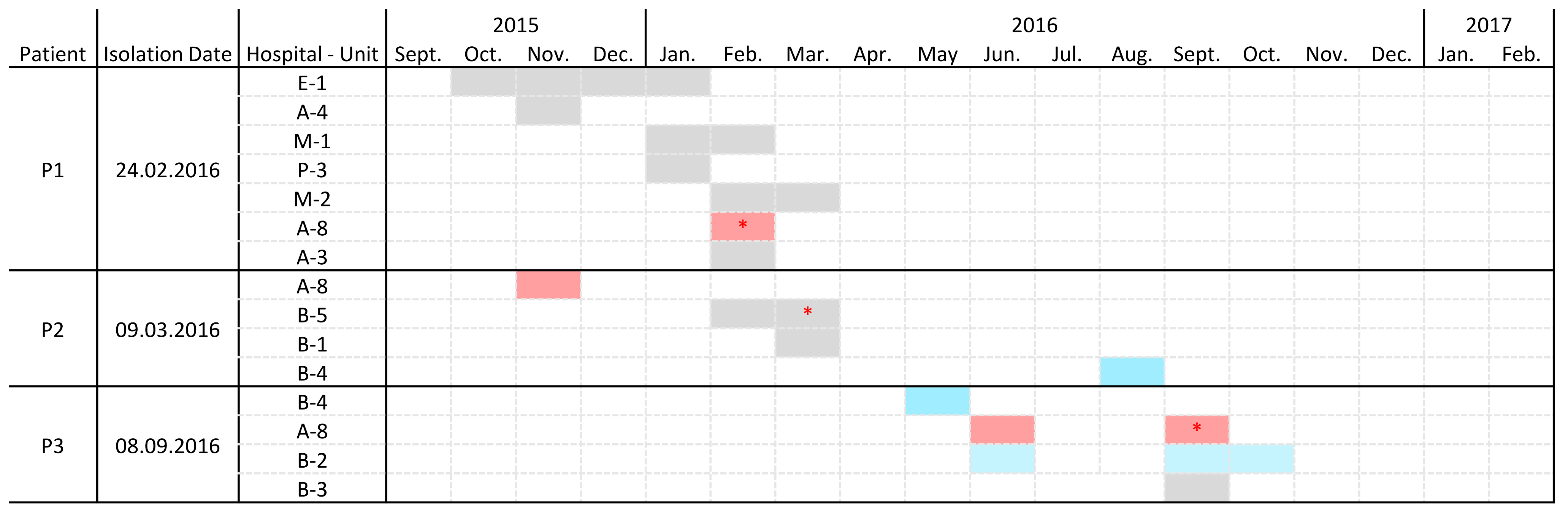

Supplement: FIG S6 [file sph004182593sf6.tif]

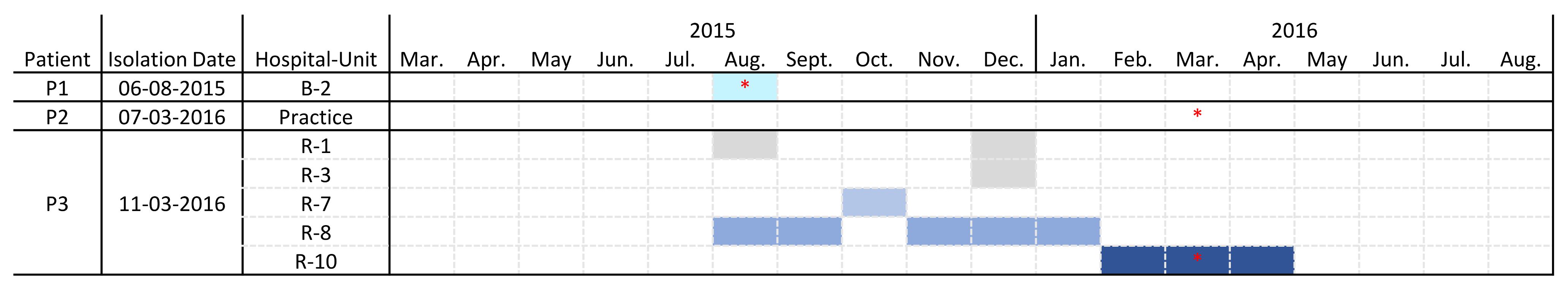

Supplement: FIG S7 [file sph004182593sf7.tif]

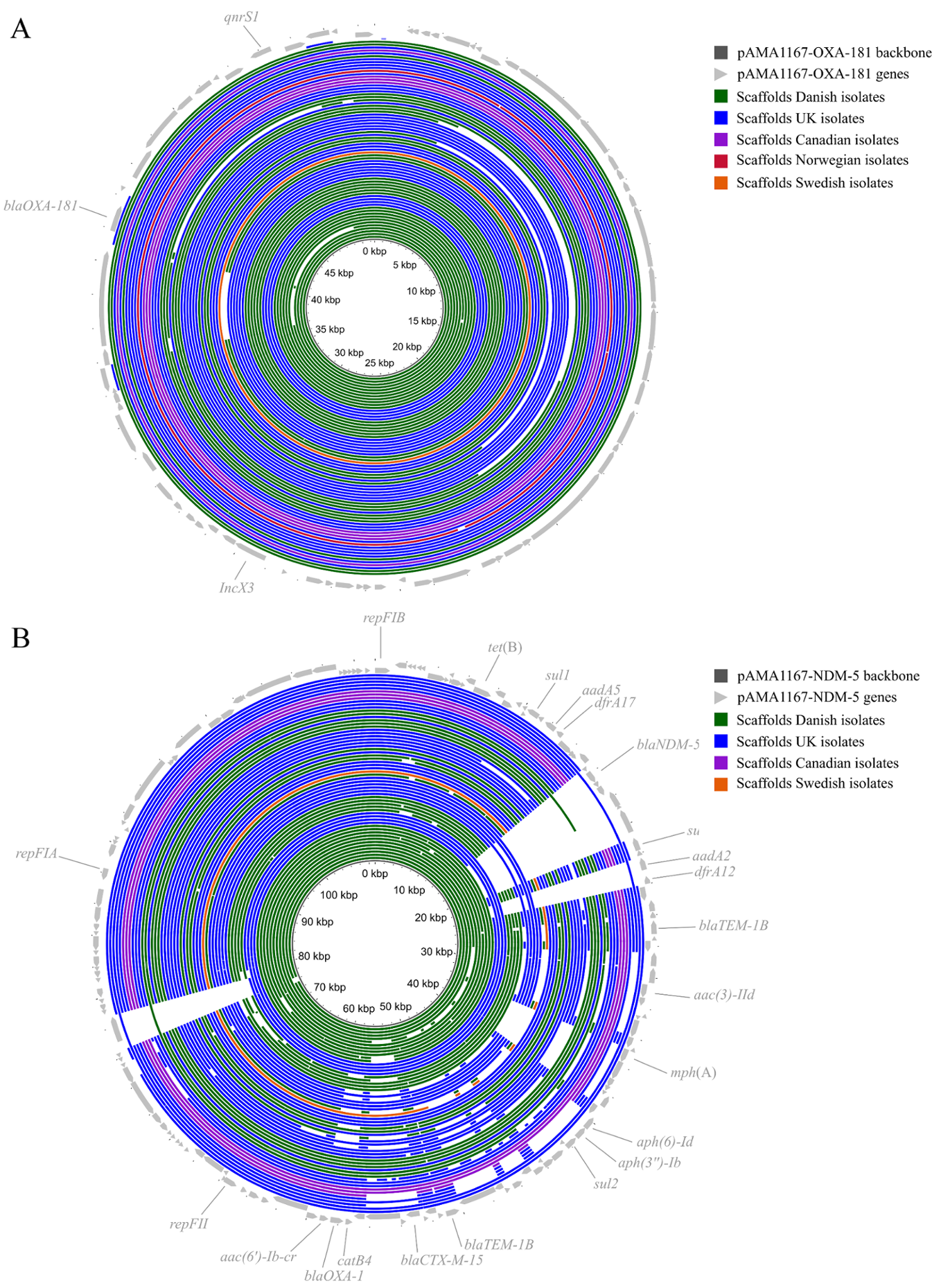

Supplement: FIG S8 [file sph004182593sf8.tif]

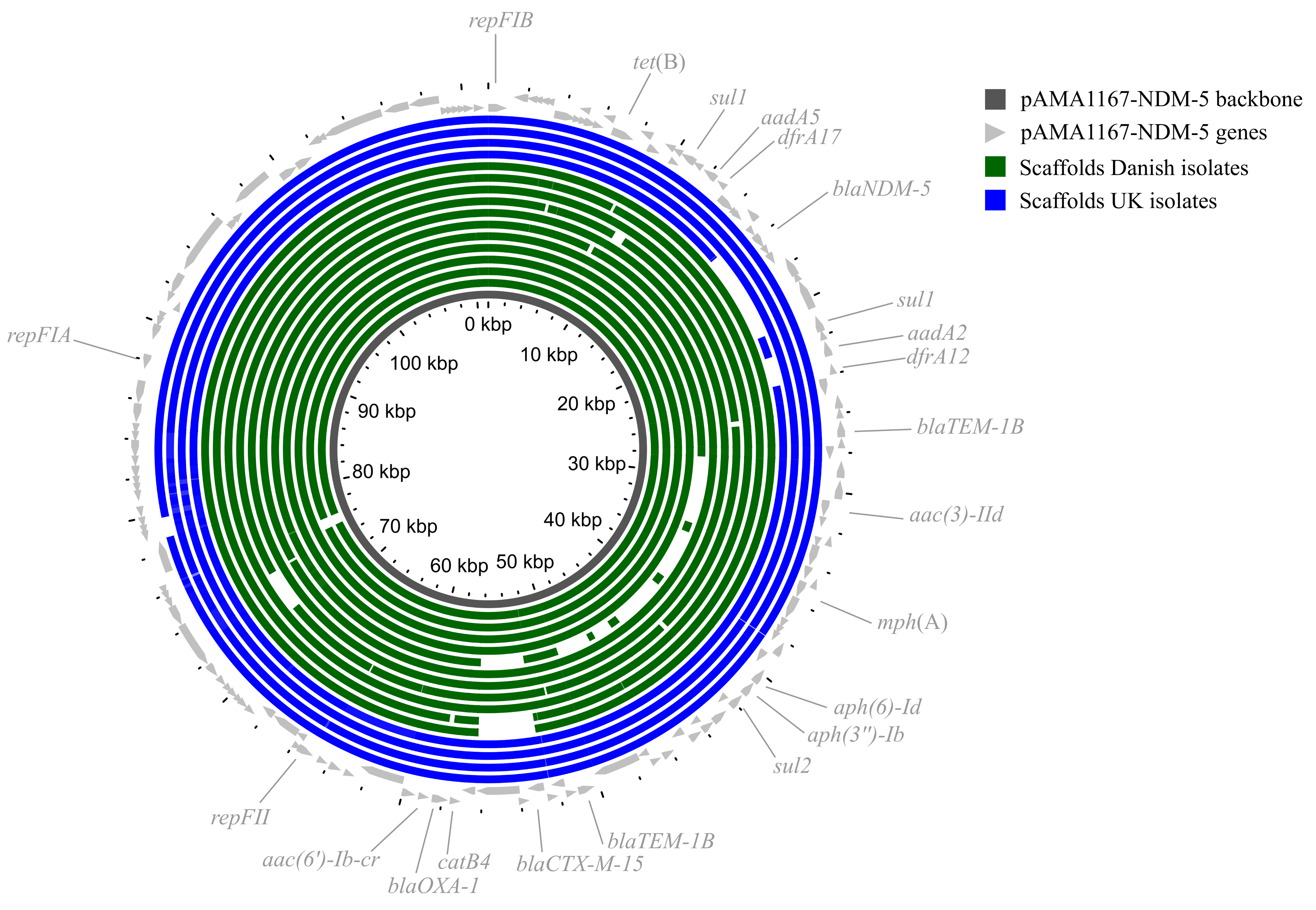

Supplement: FIG S9 [file sph004182593sf9.tif]
